# Oropharyngeal Carcinoma with a Special Focus on HPV-Related Squamous Cell Carcinoma

**DOI:** 10.1146/annurev-pathmechdis-031521-041424

**Published:** 2023-01-24

**Authors:** Robert L. Ferris, William Westra

**Affiliations:** 1 UPMC Hillman Cancer Center, Pittsburgh, Pennsylvania, USA;; 2 Department of Pathology, Icahn School of Medicine at Mount Sinai Hospital, New York, New York, USA

**Keywords:** human papillomavirus, head and neck squamous cell carcinoma, oropharynx, nonkeratinizing, immune escape

## Abstract

Human papillomavirus–positive oropharyngeal squamous cell carcinoma (HPV-OPSCC) has one of the most rapidly increasing incidences of any cancer in high-income countries. The most recent (8th) edition of the Union for International Cancer Control/American Joint Committee on Cancer staging system separates HPV-OPSCC from its HPV-negative counterpart to account for the improved prognosis seen in the former. Indeed, owing to its improved prognosis and greater prevalence in younger individuals, numerous ongoing trials are examining the potential for treatment deintensification as a means to improve quality of life while maintaining acceptable survival outcomes. Owing to the distinct biology of HPV-OPSCCs, targeted therapies and immunotherapies have become an area of particular interest. Importantly, OPSCC is often detected at an advanced stage, highlighting the need for diagnostic biomarkers to aid in earlier detection. In this review, we highlight important advances in the epidemiology, pathology, diagnosis, and clinical management of HPV-OPSCC and underscore the need for a progressive understanding of the molecular basis of this disease toward early detection and precision care.

## 1. INTRODUCTION

Approximately 15,000 cases of human papillomavirus–positive (HPV+) oropharynx cancer (OPC) are diagnosed annually in the United States and have superior cure rates to tobacco- and alcohol-associated OPC (1, 2). HPV^+^ OPC commonly presents with a small primary tumor and cervical lymphadenopathy amenable to surgical treatment (3, 4). Transoral robotic surgery or transoral laser microsurgery may address this malignancy in a minimally invasive manner (5) and, when combined with risk-adjusted postoperative radiation therapy, may achieve comparable cure rates. Retrospective reports suggest improved functional results with primary transoral surgery (TOS) (6, 7), yet its role in multidisciplinary management and treatment deintensification remains uncertain.

Treatment either with surgery and adjuvant therapy (8) or with definitive chemoradiotherapy (CRT) achieves high rates of cure for HPV^+^ OPC (1).However, as patients are likely to live longer and experience the associated long-term toxicity of definitive CRT (9), there is interest in radiation and/or chemotherapy deintensification for those patients with excellent prognosis. Both surgical and nonsurgical deintensification strategies are actively being pursued in numerous clinical trials (10, 11). TOS with de-escalated postoperative management is one potential deintensification strategy (6, 12). Retrospective studies of TOS compared with definitive CRT suggest high oncologic efficacy and improved functional results in patients undergoing surgery, with decreased gastrostomy tube dependency (6, 7, 13).

## 2. THE HUMAN TONSIL

### 2.1. Anatomy and Microanatomy

The oral cavity and oropharynx form one continuous chamber lined by an uninterrupted stratified squamous epithelium, and those carcinomas arising from this epithelial lining have long been regarded as a monotonous tumor type. Accordingly, the historical convention has been to consolidate these sites together under the term oral and their neoplastic derivatives as oral cancer. This persistent disregard for the anatomical, histological, ultrastructural, and immunological variances, however, has masked important differences in incidence trends and clinical outcomes that only become evident once the oral cavity and oropharynx are partitioned. For example, recent analyses of cancer registry data show dramatic increases in the incidence of oropharyngeal carcinomas during the past 15–20 years as the incidence of oral cavity carcinomas has remained constant or declined (14). As for clinical research, meaningful comparison of treatment responses for patients enrolled in therapeutic trials can now be made only when careful attention is given to tumor site. To remedy this long-standing limitation, recent updates in the staging and classification of head and neck tumors now appropriately divide squamous cell carcinomas of the oral vault into tumors of the oral cavity and oropharynx (15). The most notable discriminating feature between these sites is the presence of tonsillar tissue (i.e., lingual and palatine tonsils) in the oropharynx and its absence in the oral cavity. Differences in the epidemiology, pathology, and clinical behavior of squamous cell carcinomas are dramatically influenced by whether they arise from the tonsillar or nontonsillar bearing regions of the oral vault, reflecting the finding that squamous cell carcinoma caused by HPV consistently takes origin from the tonsillar tissue of the oropharynx, while squamous cell carcinomas unassociated with HPV consistently arise from the surface epithelium lining nontonsillar sites. Even within the region of the oropharynx, the distribution of HPV-related cancers is not uniform. Precision mapping of HPV-driven tumorigenesis in the oropharynx suggests that this conventional way of partitioning the oropharynx and oral cavity, however, may be too inexact. When oropharyngeal squamous cell carcinomas (OPSCCs) are further stratified on the basis of the presence or absence of tonsillar tissue, only 3% of OPSCCs arising from nontonsillar sites were found to harbor high-risk HPV compared with 92% of OPSCCs arising from the lingual and palatine tonsils (16). This observation prompts consideration of further anatomic subtyping of the oropharynx and oral cavity on the basis of the presence or absence of tonsillar tissue.

**Figure 1:**
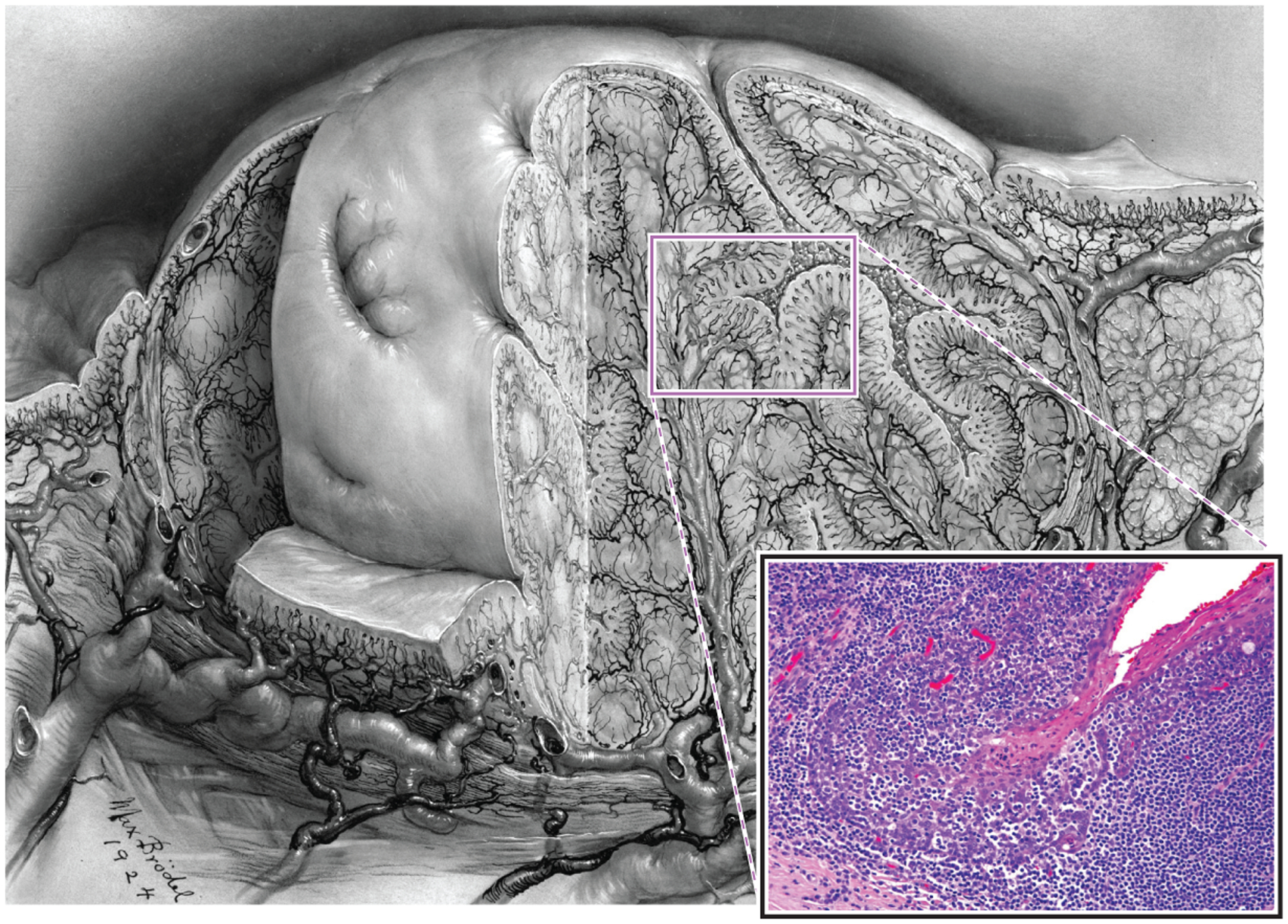
Drawing of tonsil illustrating numerous branching crypts (original drawing by Max Brödel; provided with permission by the Department of Art as Applied to Medicine at The Johns Hopkins Hospital). Inset shows the histologic features of the reticulated epithelium lining the crypts where the basaloid squamous cells are obscured by infiltrating lymphocytes.

**Figure 2:**
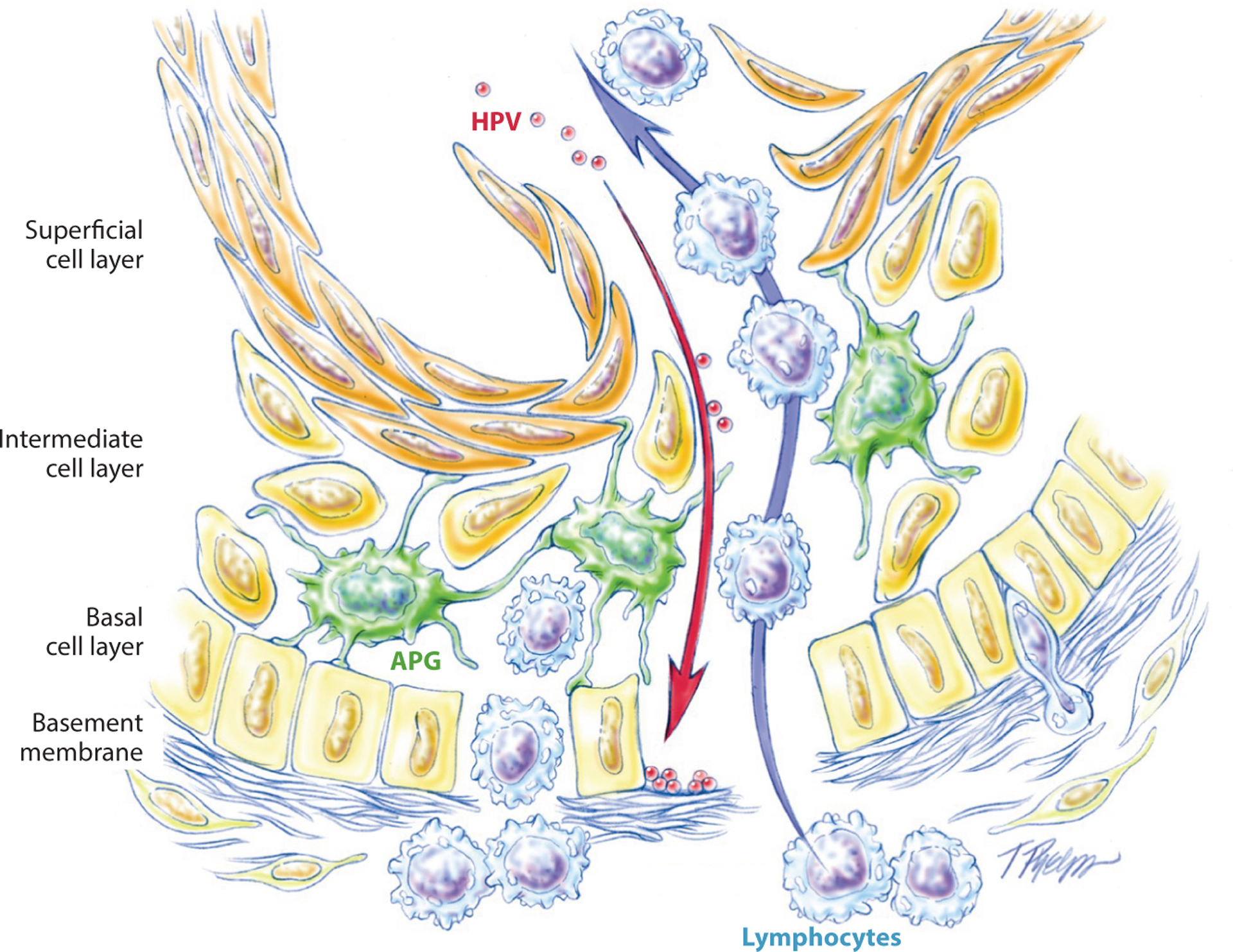
Schematic rendition of the tonsillar crypt epithelium highlighting a disrupted basement membrane, a porous epithelial-subepithelial interface, and a thin and disrupted epithelium permitting exposure of the basement membrane to viral particles. Drawing by T. Phelps; reproduced from Reference 105. Abbreviation: HPV, human papillomavirus.

The tonsil is a nonencapsulated structure that forms Waldeyer’s ring—a circular sheath of lymphoepithelial tissue that guards the opening of the upper aerodigestive tract and serves as a first line of defense against airborne and ingested antigens. The tonsils of the oropharynx include the palatine tonsils that are situated laterally in the oropharynx within the triangular tonsillar fossa and the lingual tonsil that resides on the dorsum of the posterior tongue between the sulcus terminalis and the vallecula. Ectopic tonsillar tissue has been described in other head and neck sites (e.g., floor of mouth, ventral tongue, larynx, hypopharynx) representing potential nonoropharyngeal sites for HPV-targeted carcinogenesis (17). The surface area for antigen processing is expanded more than 700-fold by the presence of numerous branching crypts that extend through the full thickness of the palatine and lingual tonsils (Figure 1) (18). These tonsillar crypts are lined by a highly specialized epithelium known as the reticulated (net-like) epithelium that is uniquely structured to facilitate transport of foreign antigens from the external environment of the oropharynx to the tonsillar lymphoid tissue (Figure 1). The basal cell layer is incomplete and its supporting basement membrane is disrupted and porous, thus allowing for the direct passage of lymphocytes and antigen-presenting cells. The intermediate layer is permeated by lymphocytes and antigen-presenting cells, and the superficial layer is thin and fragile. Complete desquamation of the superficial cells exposes the internal environment of the tonsil to external pathogens (Figure 2).

The reticular epithelium may be well suited for its role in mucosal immune protection, but those same structural features render it vulnerable to attack by HPV. In the cervix, HPV infection requires disruption (e.g., mechanical abrasion) of the epithelium with subsequent deposition of the virus onto an exposed basement membrane (19). In the reticulated epithelium of the tonsil, epithelial disruption and unrest leave the basal cell layer and the basement membrane exposed to viral deposition without the need for mucosal trauma (Figure 2). The subsequent steps culminating in malignant transformation of the HPV-infected basal cells are not well understood, but the microanatomy of the crypt epithelium may contribute to certain clinical characteristics of HPV-related tonsillar cancer such as the propensity of even small carcinomas to present as advanced regional metastases (20, 21). Origin from the deeply penetrant crypts conceals the presence of premalignant lesions, and discontinuity of the basement membrane may facilitate early invasion and metastasis of occult cancers.

### 2.2. Role of Immune Evasion in HPV Persistence and Malignant Transformation of the Tonsillar Epithelium

An understanding of the HPV replication cycle is paramount to understanding how the virus evades the immune system to establish chronic infection. HPV infects cells in the basal layers of squamous epithelia, where its early gene products are kept at low copy numbers. The viral DNA is then replicated such that the viral copy number is approximately 50–100 per host cell (22). The viral DNA is then kept as an intracellular episome, during which the oncoproteins E6 and E7 are kept at low expression levels (22). Once the infected host cell begins to differentiate into a keratinocyte, there is substantial viral (23) replication and upregulation of viral genes, including E6 and E7. This process can be amplified if the episomal viral DNA is integrated into the host genome, interrupting the E2 gene, which normally represses the expression of E6 and E7 (24). The virus then expresses its capsid proteins, L1 and L2, and viral assembly occurs just prior to shedding of the mature keratinocyte from the epithelium.

Several aspects of this life cycle allow human papillomaviruses to escape detection by the immune system. First, the virus is maintained at low copy numbers in the basal layer where immune surveillance occurs. Expression of viral DNA at high copy number, or of the highly immunogenic capsid proteins, does not occur until the virus reaches the superficial, differentiated layers of the epithelium where immune cells are scarce (24, 25). Second, the virus is shed along with desquamated keratinocytes. Without induction of host cell lysis and an associated inflammatory response, there is no stimulation of the adaptive immune response (22–24, 26, 27). The absence of any blood-borne phase in HPV infection further limits exposure of the virus to immune cells (22, 24), as does its limited access to lymphatics and lymph nodes (23). In essence, HPV has evolved to become virtually invisible to host immune cells (23, 26).

The resident monocytes of the reticulated epithelium may foster a permissive microenvironment that facilitates HPV infection and persistence (28). As one example, the immune checkpoint ligand localized expression of the immune checkpoint ligand programmed death ligand 1 is strongly expressed within the crypts (29, 30). Immune checkpoint pathways are further discussed below.

## 3. HPV AS AN ETIOLOGIC AGENT IN OPSCC

The detection of HPV in head and neck squamous cell carcinoma (HNSCC) dates back to the mid-1980s (31), but its role in the initiation and maintenance of malignancy was not decisively established until 2000 when evidence began to mount confirming its nature as a causal agent (32, 33). The biologic plausibility of HPV as a carcinogen of human epithelia is deeply rooted in an extensive experience with cervical cancers where substantial evidence has led to the conclusion that HPV is an important etiologic agent. Like cervical cancer, epidemiologic studies have observed a consistent association between HPV exposure risk and HPV-HNSCC (2, 34). Studies documenting HPV integration into the host genome, expression of viral mRNA transcripts, translation of viral oncoproteins, and disruption of key tumor suppressor pathways support the biologic activity of HPV and dismiss the notion of passenger HPV that is coincidental to tumor development (32). Indeed, the consistent presence of HPV across all stages of clinical progression underscores its obligatory role in both the initiation and maintenance of the malignant phenotype (35). Disruption of the p53 and retinoblastoma (Rb) pathways by the viral oncoproteins E6 and E7 precludes the need for various genetic alterations induced by chronic cigarette exposure, thus giving HPV-OPSCC a molecular-genetic profile that is quite distinct from smoking-related cancers (36, 37). The presence of HPV in OPSCC also marks a clinically distinct form of HNSCC characterized by improved clinical outcomes relative to its HPV-negative (HPV^−^) counterpart (1, 38). In effect, HPV is an important causative agent in a subset of HNSCCs, and its recognition amounts to nothing less than the identification of a pathologically, genetically, and clinically distinct tumor entity.

HPV is a small, nonenveloped, double-stranded DNA virus that infects squamous epithelia (25). Its genome includes early genes E1 and E2, which are replication factors; E5, E6, and E7, which are oncoproteins that disrupt cell cycle regulation in the host cells; and E4, an additional gene expressed early in the HPV life cycle whose function is not well understood. The late genes L1 and L2 encode the viral capsid proteins (25, 39). The oncoproteins E6 and E7 contribute to oncogenesis in part via functional inactivation of the cell cycle regulators p53 and Rb, respectively. The majority of HPV^+^ OPSCC (HPV-OPSCC) involves the high-risk subtype HPV alpha type 16 (HPV 16); E6 and E7 of high-risk, oncogenic subtypes tend to bind p53 and Rb with greater affinity than E6/E7 of low-risk subtypes (40). As discussed below, the expression of these genes at different points in the viral life cycle constitutes one mechanism by which HPV is able to evade the immune system of the host.

Of the more than 200 known genotypes of HPV, HPV 16 dominates the oncologic landscape (41). HPV 16 is the dominant HPV genotype detected in OPSCC, but it is not the only genotype detected (42). In one large clinical experience of 637 OPSCCs, HPV 16 was identified in 88% of theHPV^+^ cancers, followed by types 35 (5%), 33 (4%), 18 (1%), and others (2%) (43). Importantly, this distribution does not mirror the HPV distribution reported in other sites such as the cervix (44). The differential pattern of HPV genotype distribution likely reflects complex interactions between viral exposures, modes of transmission, tissue susceptibilities, and other factors that remain to be delineated. Accordingly, genotype-specific strategies for the diagnosis, treatment (e.g., HPV therapeutic vaccines), and surveillance of HPV-OPSCC should be customized according to site-specific distribution rather than inappropriately modeled from the cervical cancer experience. Virtually everything known about the clinical behavior of HPV-OPCC is dominated by HPV 16–related cases and assumes clinical equivalency across the different HPV genotypes. Results from the few studies that have addressed the survival impact of specific HPV genotypes have been mixed, with some suggesting that non-16 variants may be associated with worse outcomes (45, 46) and others showing similar survival outcomes (42, 47).

## 4. EPIDEMIOLOGY OF OPSCC

OPSCC comprises cancers of the tonsils, base of tongue, soft palate, and uvula. Like other HNSCCs, OPSCC has historically been linked to alcohol and tobacco consumption. A reduction in the prevalence of smoking in most high-income countries over the past 20 years has led to a decline in the incidence of HNSCC; however, carcinogenic HPV infection has emerged as an important risk factor that has driven an increase in the incidence of OPSCC over the same period. More specifically, HPV now accounts for 71% and 51.8% of all OPSCCs in the United States and the United Kingdom, respectively (48–51). Of these, 85–96% are caused by HPV 16 infections and are therefore expected to be preventable by prophylactic HPV vaccination, which is known to be effective in preventing HPV-associated cervical neoplasia and is now being administered to both boys and girls in several countries (51, 52).

Among all cancers, OPSCC has one of the most rapidly rising incidences in high-income countries (2, 53). An increasing incidence of this disease has been observed in the United Kingdom, in the United States, across Europe, in New Zealand, and in parts of Asia (2, 38, 54–62). In both the United Kingdom and the United States, the incidence of OPSCC cancer in men has surpassed that of cervical cancer in women (53). Globally, the percentage of OPSCCs that are HPV^+^ was reported in 2021 to be 33%; however, prevalence varies considerably depending on the geographical region, with estimates ranging from 0% in southern India to 85% in Lebanon (63). HPV-OPSCC is more prevalent than HPV^−^ OPSCC among those who do not consume tobacco or alcohol (53); however, a substantial history of tobacco and alcohol use remains prominent in patients with the former and is associated with worse outcomes (64, 65). Furthermore, sexual behavior is an established risk factor for HPV-OPSCC, with a strong association observed between number of lifetime oral sex partners and incidence of the disease (49, 66). This association might partially reflect an observed gender disparity, given that men are more likely than women to report increased numbers of sexual partners (67). Risk of oral HPV infection is associated with an increased number of recent (within the past 3 months) oral and vaginal sex partners (67).

The incidence of both HPV^+^ and HPV^−^ OPSCC has increased over the past two decades, although evidence suggests that the former is increasing more rapidly. In Denmark, a threefold increase in HPV-OPSCC was observed between 2000 and 2017, compared with a twofold increase in HPV^−^ OPSCC (57). Likewise, in Taiwan, HPV-HNSCC is rising more rapidly than HPV^−^ HNSCC, particularly those cancers arising in the tonsils (55). In Italy, the incidence of HPV-OPSCCs (as a percentage of all OPSCCs) increased from 16.7% in 2000–2006 to 46.1% in 2013–2018 (58). Lower- to middle-income countries in southern Asia and sub-Saharan Africa bear the vast majority of the global burden of HPV-associated cervical cancer, although epidemiological reports that focus on HPV-OPSCC are scarce and, as a result, whether similar rising trends are absent or are simply thus far undetected in these regions remains unclear (68). From the few reports available, the prevalence of HPV-OPSCCs in sub-Saharan Africa is low, with very few cases reported to date despite high rates of HPV-associated cervical cancer (69–72). In an investigation of HPV-OPSCC in Mozambique, the authors proposed that the low prevalence of HPV-OPSCC in their cohort (14.5%) might reflect the limited practice of oral sex in the region. This observation has been reiterated by other investigators, who observed low rates of oral HPV infection among HIV-infected individuals in northwest Cameroon and attribute this, at least in part, to limited oral sexual behaviors relative to higher-income countries (70).

Historically, most HPV-OPSCCs occur in men, which might reflect differences in both susceptibility and infection transmissibility through sexual activities, although this has yet to be fully elucidated (51, 73–75). However, an increase in incidence has been observed among White women in the United States (74). In a recent meta-analysis including data from 12 studies, the authors observed a similar prevalence of HPV-driven OPSCCs in both males and females, despite most of the assessed patients with OPSCC being male (76).

The prevalence of HPV-OPSCC was previously reported to decrease with increasing age; however, the burden of disease has begun to shift toward older men as a result of a birth cohort effect (51, 75). In one study, the median age at diagnosis increased from 53 to 58 years of age between 1998 and 2013 (77), while another study reported a similar increase, from 52 to 59 years of age between 2002 and 2017 (78). A rapidly increasing incidence in White men ≥65 years of age has also been observed, and nearly 10% of cases have been reported in those ≥70 years of age (74, 78). Nevertheless, increased rates of HPV-OPSCC continue to be evident in both younger and older adults and, although the burden is shifting toward older adults, most cases remain in those <65 years of age (73, 79, 80).

In the United States, a higher prevalence of HPV-OPSCC has been observed in White individuals than in those of other ethnicities (81, 82). In an analysis of the Surveillance, Epidemiology and End Results database, a significant increase in rates of oropharyngeal cancer in men of White or Hispanic ethnicity, and in men of other ethnicities, was observed, albeit with a decrease in Black men (74). However, another report describes a significantly more rapid increase in the prevalence of HPV-OPSCC in Black and Hispanic American individuals compared with White American individuals (2, 74). These results might reflect a greater decrease in HPV^−^ cancers in Black men compared with White and Hispanic men, resulting in the observed relative increase in HPV^+^ cancer; however, this suggestion has yet to be confirmed. In parallel with the increased incidence in White men in the United States, higher socioeconomic status is also associated with an increased incidence of HPV-OPSCC (82).

Importantly, most studies of the epidemiology of HPV-OPSCC have been conducted in the United States and are therefore not necessarily generalizable to other parts of the world, where differences in culture and behavior might influence the various lifestyle factors that have a role in the etiology of HPV-OPSCC. Further studies in geographically diverse, and particularly non-Western, regions are needed to inform region-specific guidelines, particularly with regard to clinical management and targeted public health measures.

### 4.1. Histopathology of HPV-OPSCC

Histopathologic evaluation of HNSCC is necessary to establish accurate diagnosis and may show varying features and characteristics between HPV^+^ and HPV^−^ HNSCC.

#### 4.1.1. HPV-OPSCC, typical form.

HPV-OPSCC preferentially targets the highly specialized reticular epithelium lining the tonsillar crypts. Involvement of the tonsillar surface, when it occurs, is generally a secondary phenomenon reflecting colonization of the surface epithelium as the carcinomas spill over from the tonsillar crypts. The transition between HPV-HNSCCs and the adjacent surface epithelium tends to be abrupt without transitional zones of epithelial precursor lesions (i.e., squamous dysplasia). Indeed, the histologic progression through the sequential stages of dysplasia culminating in carcinoma in situ and invasive growth that characterize non-HPV-HNSCCs is not generally evident for HPV-OPSCCs. The inability to histologically characterize the early stages of HPV-induced tumorigenesis continues to deter efforts to diagnose precancerous lesions and assess cancer risk.

As these carcinomas infiltrate, they tend to invade as sheets, lobules, or ribbons of cells. Central necrosis within expanding tumor lobules, sometimes giving rise to cystic degeneration, is a frequent finding. Invasive growth often does not elicit a strong desmoplastic stromal reaction. Instead, the tumor nests are often surrounded by a zone of lymphoid cells that permeate the tumor nests as tumor-infiltrating lymphocytes. In the typical tumor, the tumor cells display a high nuclear-to-cytoplasmic ratio, oval to spindled nuclei, and syncytial cytoplasm (indistinct cell borders) without intercellular bridges and usually lack significant cytoplasmic keratinization (66). These cellular features may impart a distinct basaloid appearance. Occasionally, tumors exhibit partial or full keratinization such that the distinction between HPV-dependent and HPV-independent OPSCC should not be based on keratinization alone. Even though the tumor cells closely resemble the reticulated epithelium from which they arise, they are often erroneously regarded by pathologists as poorly differentiated or even undifferentiated carcinomas on the basis of their basaloid appearance and the absence of keratin production. To circumvent this common problem, histologic grading is no longer advocated for HPC-OPSCC (49).

In lymph node metastases, the presence of cystic degeneration is a common finding in HPV-OPSCCs, and its presence should warrant strong consideration of a metastasis from the tonsil. These squamous lined cysts of the lateral neck are sometimes clinically and histologically mistaken for branchial cleft cysts (67).Fine-needle aspiration (FNA) of the cyst contents gives rise to highly degenerated specimens consisting of macrophages and necrotic debris. A definite cytopathologic diagnosis may not be possible if the aspirate does not incorporate the cyst lining.

#### 4.1.2. HPV-OPSCC, variant forms.

Subsets of HPV-HNSCC deviate from the morphologic prototype, and these are set apart as morphologic variants of HPV-related HNSCC. To date, these variants include papillary squamous cell carcinoma (68), adenosquamous (69) [including a ciliated form (27)], basaloid (28), lymphoepithelial-like (29), sarcomatoid (83), and high-grade neuroendocrine (73–75). With the notable exceptions of HPV-related small cell carcinoma and large cell neuroendocrine carcinoma (see below), morphologic variance does not seem to influence clinical behavior. A few of these variants warrant further comment due to their propensity to be confused with other subtypes of HNSCC.

##### 4.1.2.1. Basaloid variant.

In the basaloid variant of HPV-OPSCC, a lobular arrangement of compact tumor cells with a high nuclear-to-cytoplasmic ratio in the absence of keratinization conveys a distinctly basaloid appearance. When the basaloid morphology is highly developed, HPV-related squamous cell carcinoma may be histologically indistinguishable from the basaloid variant of non-HPV-related squamous cell carcinoma—a variant of HNSCC that is set apart by its striking basaloid morphology and its aggressive clinical behavior. Morphologic similarities aside, HPV-OPSCCs do not share the same aggressive clinical behavior that characterizes the basaloid variant of squamous cell carcinoma (71, 76). In a squamous cell carcinoma with highly developed basaloid features, the presence of HPV is significantly associated with improved overall survival (OS).

##### 4.1.2.2. Lymphoepithelial variant.

Some HPV-related squamous cell carcinomas demonstrate lymphoepithelial features including tumor cells with syncytial cytoplasm, vesicular nuclei, and large central nucleoli dispersed in an inflammatory background as cell clusters or single cells (72, 77). When these lymphoepithelial features are highly developed, an HPV-HNSCC may be mistaken for an Epstein-Barr virus (EBV)-induced undifferentiated carcinoma of the nasopharynx. On the basis of this morphologic overlap, one cannot assume an EBV-driven process by phenotype alone. Failure to recognize that the lymphoepithelial phenotype is not restricted to nasopharyngeal carcinomas may be particularly problematic when these features are encountered in cervical lymph node metastases. Assumptions about nasopharyngeal origin based solely on the morphologic findings run the risk of inappropriately diverting treatment away from the oropharynx and toward the nasopharynx. Accordingly, testing for both HPV and EBV is advisable when lymphoepithelial carcinomas are encountered as lymph node metastases in patients with occult primary tumors.

##### 4.1.2.3. HPV-related high-grade neuroendocrine.

Several reports have underscored the presence of HPV-related carcinomas of the head and neck characterized by well-developed features of small cell carcinoma (33) or large cell neuroendocrine carcinoma (74). The small cell variant is composed of small anaplastic cells with hyperchromatic nuclei and scant cytoplasm. Large cell neuroendocrine carcinomas are composed of medium to large polygonal cells with abundant cytoplasm, coarse to vesicular chromatin, prominent nucleoli, and nuclear palisading around the periphery of the tumor nests. Both forms of high-grade neuroendocrine carcinoma demonstrate immunohistochemical evidence of neuroendocrine differentiation. Importantly, HPV does not appear to convey a favorable prognosis when its presence is detected in this small cell variant. HPV-related high-grade neuroendocrine carcinomas of the oropharynx appear to share the same aggressive clinical features of their counterparts in the lung where a high-grade neuroendocrine phenotype is associated with early distant spread and poor OS (75, 78). Consequently, HPV-related carcinomas of the oropharynx showing a high-grade neuroendocrine phenotype should be regarded as a poorly/undifferentiated form of HPV-related oropharyngeal carcinoma where tumor morphology supersedes HPV positivity as a prognostic indicator. Recently, HPV-associated small cell carcinoma of the head and neck has been strongly associated with the HPV 18 genotype (64).

### 4.2. HPV Detection

Since HPV prevalence varies between subsites of cancers of the head and neck and across different regions of the world, reliable and appropriate detection methods are crucial to effectively determine etiology of HNSCC.

#### 4.2.1. When to test for HPV.

Routine testing of head and neck squamous cell carcinoma is informed by the site of tumor origin. For those carcinomas arising in the oropharynx, HPV detection is well established as a powerful biomarker indicating a more favorable clinical outcome such that routine HPV assessment has become standard pathology practice for the evaluation of all OPSCCs. Indeed, the College of American Pathologists, the American Joint Committee on Cancer, and the National Comprehensive Cancer Network have all recommended routine HPV testing as part of the standard pathologic evaluation of OPSCCs for the purpose of diagnosis and molecular tumor staging (1, 64). The finding of HPV in up to 20% of sinonasal carcinomas may support increased testing at this site as part of an investigational process, but expanding the scope of routine diagnostic HPV testing to nonoropharyngeal sites is not warranted until studies establish a clear relationship between HPV infection and a distinct clinical behavior including treatment responses. At sites that are not preferentially targeted by HPV such as the oral cavity, larynx, and hypopharynx, the likelihood that a positive HPV test (e.g., p16 immunohistochemical staining) truly reflects the presence of transcriptionally active and clinically relevant HPV infection may be unacceptably low (i.e., poor positive predictive value). Furthermore, a true positive test does not provide compelling evidence of a distinctly unique form of HNSCC that differs from its HPV^−^ counterparts when dealing with a nonoropharyngeal site. For patients with HPV^+^ HNSCCs arising in nonoropharyngeal sites, risk factor profiles and clinical outcomes more closely resemble smoking-related HPV^−^ carcinomas than HPV-OPSCCs (81, 82, 84).

In malignant transformation of the tonsillar epithelium, HPV does not act through a hit-and-run mechanism where its role is transient and limited to the initiation of tumorigenesis. Instead, the presence of HPV persists, and it is just as readily detected in metastatic implants as in the corresponding primary cancers. Consequently, a lymph node metastasis is quite suitable as a substrate for HPV testing, obviating the need for additional tissue acquisition in those patients with small or even occult primary cancers. For those patients who present with neck metastases in the absence of an obvious primary tumor, HPV testing of a lymph node metastasis is also an effective strategy for localizing the site of origin. In these patients, the detection of HPV in a lymph node metastasis is a reliable predictor of oropharyngeal origin (85, 86). Similarly, for the squamous cell carcinoma in the lung of a patient with a prior HPV-OPSCC, HPV detection provides compelling evidence that the tumor in the lung represents a metastasis rather than a new primary lung cancer (87, 88).

#### 4.2.2. Methods of HPV detection.

Methods of HPV testing across laboratories vary considerably, reflecting the biases and tendencies of individual investigators and the cost-to-benefit ratio of each technique. Detection strategies vary not just in design but also in their detection targets. These targets have included HPV DNA, HPV RNA, viral oncoproteins, cellular proteins, and HPV-specific serum antibodies. For widespread implementation in the clinical arena, detection methods must be accurate, technically feasible, and cost effective.

##### 4.2.2.1. p16 immunohistochemistry.

**Figure 3:**
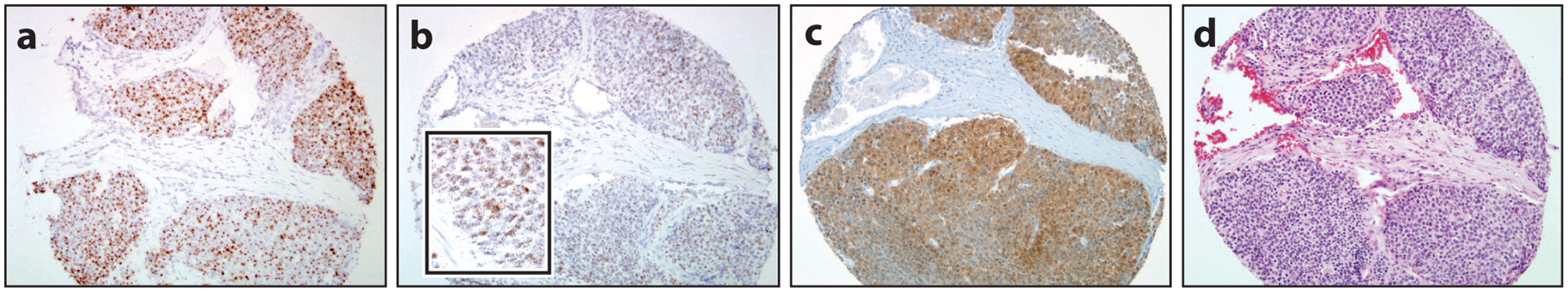
Various strategies for human papillomavirus (HPV) detection now allow for microscopic visualization of progressive steps in the cellular biology of HPV tumorigenesis from (*a*) the cellular insertion of HPV DNA (DNA in situ hybridization) to (*b*) the transcription of viral mRNA (high-risk HPV E6/E7 mRNA in situ hybridization) to (*c*) the disruption of the normal cellular machinery giving rise to high levels of expression of the cellular protein p16 (p16 immunohistochemistry). All of these steps culminate in (*d*) the prototypic microscopic appearance of HPV-related oropharyngeal squamous cell carcinoma (routine hematoxylin-eosin histology). Hybridization signals are readily apparent at higher magnification (*b*, inset). Figure adapted with permission from Reference 106; copyright 2012 Wolters Kluwer Health, Inc.

The cellular protein p16 is markedly overexpressed in tumor cells with transcriptionally active high-risk HPV. The viral E7 oncoprotein destabilizes pRb, functionally removing suppression of p16 expression and allowing tumor cells with high p16 levels to bypass pRb-dependent cell cycle arrest (89). The result is marked overexpression of p16, making it an excellent surrogate marker of viral infection in the correct context (Figure 3). Immunostaining for p16 protein has recently been regarded as a practical alternative or complementary procedure for HPV testing of oropharyngeal cancers on the basis of a high correlation between HPV detection and p16 overexpression in numerous studies. Aside from its high correlation with other detection assays, abundant literature has also established p16 immunohistochemistry (IHC) as an independent predictor of improved patient prognosis in OPSCC. On the basis of its role as a powerful independent predictor of improved patient prognosis, as well as its widespread availability, ease and reproducibility of interpretation, and excellent performance on small specimens, p16 IHC staining is currently advocated by the College of American Pathologists as the method of choice for determining HPV status of OPSCCs, at least for biopsy and resection specimens.

The mechanistic link between HPV DNA integration and p16 expression, however, is neither direct nor exclusive. The Rb gene may be inactivated by mechanisms other than E7 oncoprotein expression yet still result in high levels of p16 expression. To be truly useful as a surrogate marker of HPV infection, the interpretation of p16 IHC must be informed by various histological, anatomical, and clinical considerations (90). First, p16 IHC may substitute for HPV testing when strong staining is present in the nucleus and cytoplasm of the tumor cells throughout all or most (>70%) of the tumor. Focal or weak staining should be supported by other forms of HPV testing. Second, while the sensitivity and specificity of p16 staining as a marker of HPV infection is sufficiently high to serve as a reliable test for squamous cell carcinomas of oropharyngeal origin, these values are either unknown or unacceptably low for HNSCCs arising in nonoropharyngeal sites. Third, interpretation of p16 staining must be informed by the morphologic features of the tumor as outlined above. In those oropharyngeal carcinomas that demonstrate the typical morphology of HPV-related HNSCC, p16 IHC staining may substitute for HPV detection. Additional HPV testing should be performed in p16-negative oropharyngeal carcinomas that exhibit classic HPV-related histomorphology and in p16-positive oropharyngeal carcinomas that do not exhibit classic HPV morphology. Fourth, p16 IHC is currently used primarily as a prognostic indicator for patients with oropharyngeal carcinoma, and any expanded clinical role for HPV detection may necessitate more stringent detection methods.

##### 4.2.2.2. PCR-based methods.

Polymerase chain reaction (PCR) amplification of HPV DNA is a target-amplification technique that is capable of amplifying trace DNA sequences in a biological sample that contains heterogeneous cell types. The primer sets can be designed to target highly conserved consensus sequences shared by multiple HPV types, allowing for the simultaneous identification of a wide range of HPV types, or they can target type-specific viral DNA sequences, permitting HPV genotyping. Those who advocate PCR-based methods of HPV detection point to its incomparable sensitivity: These methods can detect HPV well below one viral copy genome per cell. The value of detecting HPV at very low levels, however, is offset by other factors that confound the biological and clinical relevance of viral detection. First, clinical samples are very prone to cross contamination by other specimens. To minimize this adverse effect, surgical pathology facilities for processing oropharyngeal and gynecologic specimens should be physically separate, and diagnostic laboratories must use meticulous PCR precautions. Second, PCR-based methods do not permit the distinction between HPV that is acting as a driver of malignant transformation and a transcriptionally silent virus that is playing no role in the process of tumorigenesis (i.e., passenger virus). The problem is highlighted in those studies that have shown significant discordance between HPVDNA detection and the actual presence of E6/E7 mRNA viral transcripts that define clinically relevant HPV infections (91). The ability to distinguish HPV infections that are clinically relevant from those that are not may be supported by a real-time PCR approach that can better measure viral load. Using this more quantitative approach, studies indicate that those tumors with a high viral load are much more likely to express E6/E7 mRNA and correlate with improved clinical outcomes (92).

##### 4.2.2.3. RNA in situ hybridization.

The ultimate goal of any developing technology for HPV detection in clinical samples is to approach the gold standard for sensitivity and specificity while maximizing efficiency, simplicity, reproducibility, and transferability to the routine diagnostic laboratory. Although the most direct and compelling evidence of HPV-related tumorigenesis is the documentation of transcriptionally active HPV in tumor cells, the detection of E6/E7 transcripts is technically challenging. The recent development of RNA in situ hybridization (ISH) probes complementary to E6/E7 mRNA now permits direct visualization of viral transcripts in routinely processed tissues. Testing for HPV E6/E7 transcripts by RNA ISH is an ideal platform for HPV detection in clinical samples. First, it confirms the presence of integrated and transcriptionally active virus by permitting the visualization of viral transcripts directly in tissue sections. Second, it is technically feasible and easily transferrable into the diagnostic pathology laboratory. Indeed, the imminent availability of the HPV RNA ISH probes method to a widely available automated staining platform promises to enhance standardization across diagnostic laboratories, decrease turnaround time for large case volumes, and improve reproducibility among clinical trials. Third, the transcription of viral mRNA provides a natural target amplification step that may dramatically improve viral detection in clinical samples and clarify the status of those perplexing tumors that are p16 positive by IHC but HPV^−^ by DNA ISH (33). Fourth, it is prognostically useful: The presence of E6/E7 mRNA transcripts is tightly coupled to the expression of other powerful prognostic markers (e.g., p16 expression) and strongly correlates with patient outcomes (93).

##### 4.2.2.4. Single-versus multimodality HPV analysis.

The power of p16 IHC staining lies in its high sensitivity for detecting all high-risk types of HPV, but it suffers from suboptimal specificity. Use of p16 staining as a stand-alone test for HPV detection is associated with a small false-positive rate where p16 expression is driven by some nonviral mechanism. These p16-positive/HPV^−^ oropharyngeal carcinomas have been associated with less favorable survival than p16-positive/HPV^+^ cancers, suggesting that selection of patients for de-escalation clinical trials may benefit from supplementary detection assays rather than p16 staining alone (94).

Multimodality detection strategies look to utilize the strengths of individual assays in combination to optimize the overall reliability of HPV detection. Current multimodality strategies utilize a stepwise approach that begins with p16 IHC staining. Those OPSCCs that are p16 positive are then analyzed with more rigorous HPV-specific detection assays such as HPV RNA ISH and/or a PCR-based assay. Although the multimodality approach may provide the most accurate analysis of HPV status, it does represent a deviation from a growing trend in HPV testing that highly values rapid turnaround, simplicity, and cost restraint. More painstaking HPV detection algorithms may be most appropriate when there is no allowance for error in determining true HPV status, such as selection of patients for de-escalation therapy or therapeutic HPV vaccine trials.

#### 4.2.3. Future directions in HPV testing.

Analysis of HPV tissue sections using surrogate biomarkers (such as p16 using IHC), versus HPV DNA or RNA detection, has various sensitivity and specificity characteristics and needs to be validated in clinical settings. Circulating tumor DNA for HPV detection is also becoming more widespread.

##### 4.2.3.1. Liquid phase assays for HPV detection in cytological samples (wet biopsy).

To date, the College of American Pathologists has not stipulated any one specific detection assay for determining HPV status in cytological specimens (95). The few studies that have addressed HPV testing of cytological samples have primarily tried to adapt tissue-targeted approaches (e.g., p16 IHC and HPV ISH) to archived cytological specimens (96, 97). In most instances, HPV testing of cytological specimens is restricted to a small subset of cases where ample cellular material is available for the construction of cell blocks. Even in these cases, p16 IHC analysis is often unreliable, such that broad-based application awaits the development of strategies that can be applied to aspirated cells without the need for high cellularity and specimen processing (98).One very promising approach that does not require the processing of cytological specimens as tissue blocks involves the use of liquid phase assays that are already in widespread use for routine assessment of cervical cancer risk. Direct transfer of cytological samples into the liquid media minimizes specimen preparation and eliminates the need for specimen processing as cell blocks. The Hybrid Capture 2 (HC2) HPV DNA test is an in vitro nucleic acid hybridization assay with signal-amplification using microplate chemiluminescence for the detection of 18 oncogenic types of HPV DNA in cervical specimens. This assay was found to correctly classify all cytological preparations when HPV status of the cytological specimens (brushings and FNAs) was compared with status of the paired surgical resection specimens (99). Similarly, the Cervista^®^ HPV test—a liquid phase assay that is clinically validated for HPV detection in cervical cytological specimens—is highly reliable in determining HPV status of cytological specimens (100). Its analytical sensitivity is comparable to that of HC2, but the addition of a housekeeping gene as an internal control to ensure sufficient cellularity diminishes the likelihood of false-negative results. In effect, HPV detection and genotyping can be achieved in cytological specimens without the need for tissue acquisition or complex specimen processing. Indeed, accurate HPV analysis can even be determined from the supernatant portion of an FNA—a rich source of DNA that is generally discarded as waste (101).

##### 4.2.3.2. Circulating HPV DNA (wet biopsy).

Cancers that are etiologically related to HPV infection present a unique opportunity for quantifying circulating amounts of HPV DNA sequences that originate in tumor tissue. Recently, a test has been developed and validated to measure circulating tumor HPV DNA in the blood using a PCR-based approach (102–104). The rate of HPV DNA clearance in the blood following chemoradiation appears to correlate with treatment sensitivity (102). Although this state-of-the-art assay has been used primarily to monitor response to therapy, it also holds promise as a tool for early detection and posttreatment surveillance for patients with HPV-OPSCC.

## 5. PROGNOSIS FOR PATIENTS WITH OPSCC

**Figure 4:**
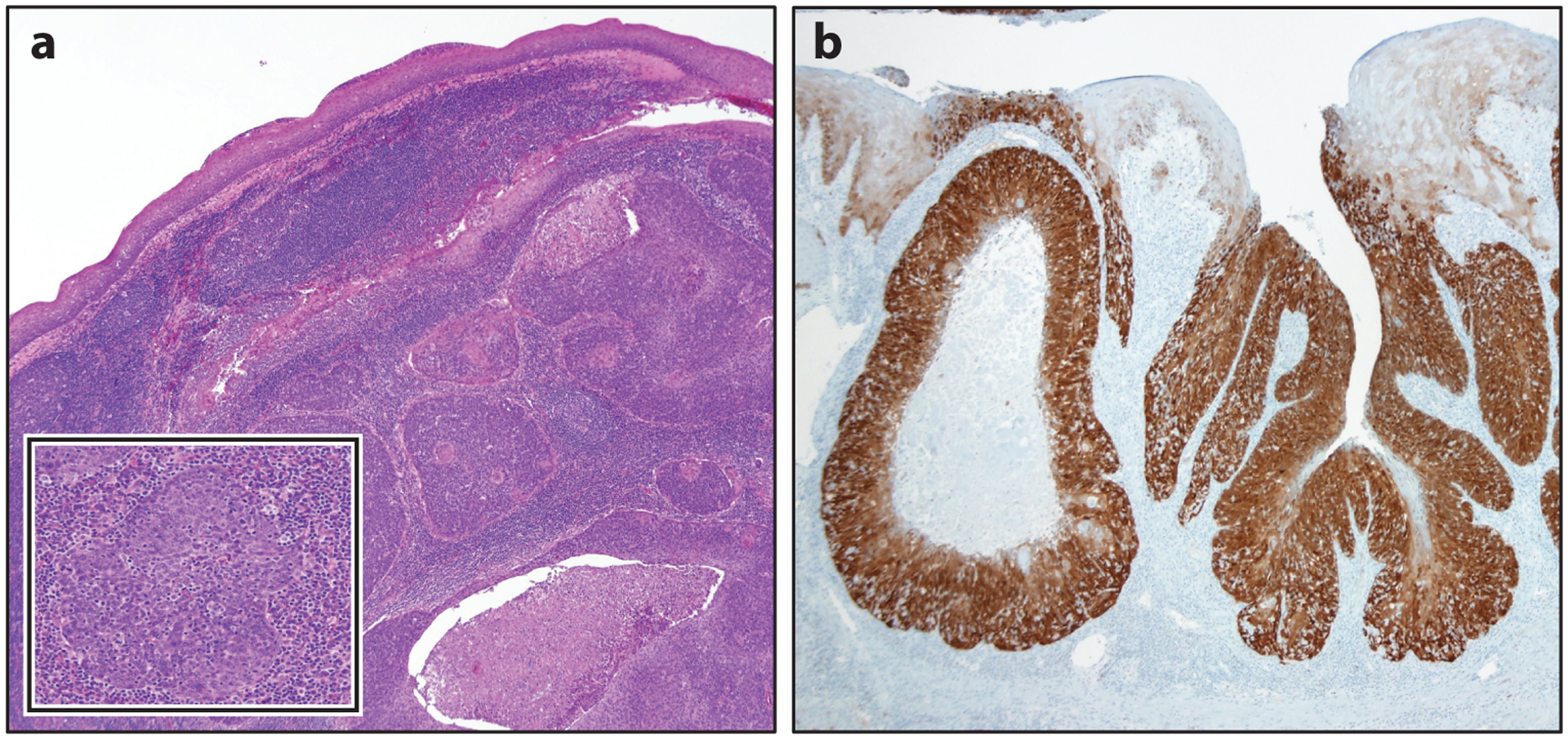
Human papillomavirus (HPV)-associated oropharyngeal squamous cell carcinomas typically arise from the tonsillar crypts and grow beneath the surface epithelium as expanding lobules, often with central necrosis (*a*). The lobules are composed of nonkeratinized basaloid cells that are surrounded and infiltrated by lymphocytes (*a*, inset). (*b*) In the reticulated crypt epithelium, p16 staining is useful in highlighting the distribution of tumor cells in the tonsils.

Converging clinical, molecular, and epidemiologic evidence now confirms that HPV status is the single most important determinant of prognosis in OPSCC. HPV is an epitheliotropic, double-stranded DNA virus with >100 characterized genotypes; HPV 16, with its predilection for oropharyngeal mucosa, is the most common genotype isolated from the oropharynx (Figure 4). HPV initiation underlies the epidemiologic observation that both incidence and survival of OPSCCs are increasing, in contrast to cancers associated with tobacco and alcohol whose incidence is decreasing with survival essentially stable (5).

**Figure 5:**
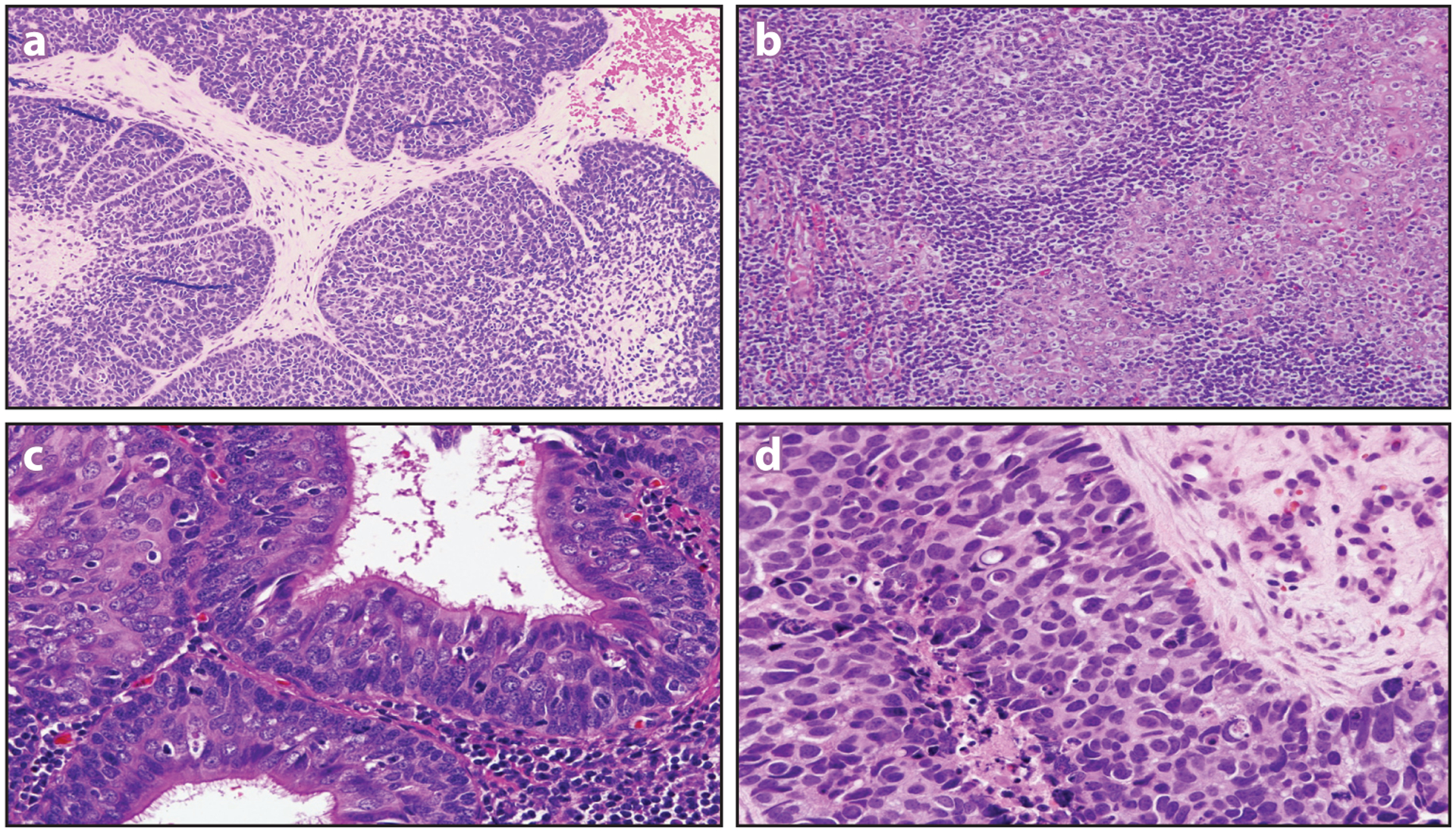
Morphologic variant forms of human papillomavirus (HPV)-associated oropharyngeal carcinoma include those that take on highly developed basaloid (*a*), lymphoepithelial (*b*), and ciliated (*c*) features. The presence of a high-grade neuroendocrine component (*d*) is a finding that signals aggressive tumor behavior.

The improved prognosis associated with HPV in oropharyngeal HNSCC is related to substantially different responsiveness to treatment. The carcinogenic process in HPV-related malignancies is primarily attributed to the viral oncoproteins E6 and E7, which bind and inactivate tumor suppressors p53 and pRb, respectively. Deficiency of p53 and Rb results in loss of cell cycle checkpoints and appropriate apoptosis. HPV-infected cells demonstrate unbridled progression through the cell cycle, a proproliferative state that benefits the HPV life cycle in early infection. HPV-related oropharyngeal malignancies more frequently appear in young, male patients with a good performance status and are associated with an early T stage, yet advanced nodal stage, often with cystic nodes (Figure 5). When compared with tumors in patients with HPV^−^ disease, HPV^+^ tumors are consistently associated with a 50% reduction in risk of death (6).This is exhibited in multiple secondary analyses of recent institutional as well as cooperative group prospective studies that examined radiation therapy (RT) alone or in combination with various chemotherapy regimens.

In the first prospective trial designed to investigate HPV-related cancers, Eastern Cooperative Oncology Group (ECOG) investigators (in the ECOG 2399 trial) utilized an induction regimen of paclitaxel and carboplatin and reported a higher response rate in those that were HPV^+^. Additionally, after a median follow-up of 40 months, progression-free survival (PFS) and OS was superior in HPV^+^ patients when compared with those that were HPV^−^. This significant response to induction therapy led to the first national cooperative group trial testing a deintensification strategy. In this recently completed trial (ECOG 1308), patients with resectable HPV^+^ oropharyngeal cancers were treated with three cycles of induction chemotherapy including cisplatin, paclitaxel, and cetuximab. Complete clinical response at the primary site was used as a dynamic response biomarker; complete responders were treated with a radiation dose reduced by 20% (54.0 Gy versus 69.3 Gy). For those receiving reduced-dose RT, PFS at 23 months was 84%, primary site local control was 94%, nodal control was 95%, and distant control was 92%. The Danish Head and Neck Cancer Group (DAHANCA) reported improved outcomes in those with HPV-related oropharyngeal malignancies. In the DAHANCA 5 trial, patients in which samples were analyzed for p16, an HPV surrogate, demonstrated improved local-regional control as well as disease-free survival after adjustment for tumor and nodal stage. Additionally, an unplanned subset analysis of the Radiation Therapy Oncology Group (RTOG) 0129 trial identified an association between tumor HPV status and survival among patients with stage III and IV HNSCC. Here, 64% of enrolled patients were HPV^+^ and were found to have improved 3-year survival (82.%) compared with the survival of those with HPV^−^ tumors (57.%) (9). Using a recursive-partitioning analysis, a significantly increased risk of death between HPV status and pack-years of smoking identified patients at low, intermediate, and high risk of death (9).With improved patient risk stratification, future trials will aim at maintaining excellent survival in the HPV^+^, nonsmoking cohort, while reducing late toxicities associated with current CRT regimens. In contrast, high-risk cohorts will be the focus of intensification strategies, as both local and distant relapse continue to affect a significant portion of patients (see Table 1).

## 6. CONCLUSIONS

Oropharyngeal squamous cell carcinoma related to latent infection with HPV is increasingly common. The presence of this virus results in a more robust immune response compared with that seen in HPV^−^ tumors. However, the virus has evolved ways to subvert innate and adaptive immunity to persist and induce malignancy. Better understanding of these mechanisms of immune evasion has resulted in the development of new, investigational immunotherapies that will ultimately enhance the efficacy of existing therapies for oropharyngeal squamous cell carcinoma.

## Figures and Tables

**Table 1:** Risk stratification in HPV-OPSCC

Risk level	Pathologic features
Low risk	Negative margins (>3 mm), N0–N1,no ENE
Intermediate risk	Close margins (< 3 mm), 2–4 positive nodes, ≤ 1 mm ENE, PNI/LVI
High risk	Positive margin, > 1 mm ENE, ≥ 5 positive nodes

Abbreviations: ENE, extranodal extension; HPV, human papillomavirus; LVI, lymphovascular invasion; N, node (value indicates degree of nodal spread); OPSCC, oropharyngeal squamous cell carcinoma; PNI, perineural invasion.
